# Mammalian cargo receptors for endoplasmic reticulum-to-Golgi transport: mechanisms and interactions

**DOI:** 10.1042/BST20220713

**Published:** 2023-06-19

**Authors:** Yuan Zhang, Vishal Srivastava, Bin Zhang

**Affiliations:** Genomic Medicine Institute, Lerner Research Institute of Cleveland Clinic, Cleveland, OH, U.S.A

**Keywords:** endoplasmic reticulum, glycoproteins, golgi apparatus, LMAN1–MCFD2, SURF4, trafficking

## Abstract

Proteins that are destined to enter the secretory pathway are synthesized on the rough endoplasmic reticulum (ER) and then translocated into the ER lumen, where they undergo posttranslational modifications, folding, and assembly. After passing a quality control system, the cargo proteins are packaged into coat protein complex II (COPII) vesicles to exit the ER. In metazoans, most COPII subunits have multiple paralogs, enabling COPII vesicles the flexibility to transport a diverse range of cargo. The cytoplasmic domains of transmembrane proteins can interact with SEC24 subunits of COPII to enter the ER exit sites. Some transmembrane proteins may also act as cargo receptors that bind soluble secretory proteins within the ER lumen, enabling them to enter COPII vesicles. The cytoplasmic domains of cargo receptors also contain coat protein complex I binding motifs that allow for their cycling back to the ER after unloading their cargo in the ER-Golgi intermediate compartment and *cis*-Golgi. Once unloaded, the soluble cargo proteins continue maturation through the Golgi before reaching their final destinations. This review provides an overview of receptor-mediated transport of secretory proteins from the ER to the Golgi, with a focus on the current understanding of two mammalian cargo receptors: the LMAN1–MCFD2 complex and SURF4, and their roles in human health and disease.

## Introduction

Compared with single-cell eukaryotes like yeast, metazoans have a much more complex secretome and corresponding secretory machinery. In metazoans, most coat protein complex II (COPII) components have multiple paralogs, providing temporal and spatial flexibility for accommodating diverse cargo during transport from the endoplasmic reticulum (ER) to the Golgi complex. Deficiencies in individual mammalian COPII paralogs have been linked to genetic diseases in humans [1–4] and various phenotypes in mice [5–12]. Two mechanisms have been proposed to explain how proteins are transported from the ER to the Golgi complex via COPII vesicles. The first is the bulk flow model, where transmembrane and soluble proteins enter COPII vesicles by random diffusion [13]. The second is the receptor-mediated transport model, where properly folded transmembrane proteins and soluble proteins that have passed quality control are actively recruited into COPII vesicles [14,15]. The SEC24 subunits of the COPII coat contain binding sites that interact directly with the cytosolic domains of transmembrane proteins [16,17], and other COPII subunits may also play a role in selecting cargoes. Some of these transmembrane proteins in turn serve as cargo receptors that interact with soluble proteins in the ER lumen, concentrating these cargoes at sites of vesicle budding. Certain transmembrane cargoes may require specific transport receptors as well [18]. Cargo receptors also contain coat protein complex I (COPI) binding motifs that direct retrograde trafficking back to the ER. Some small and rapidly secreted proteins do not seem to require cargo receptors and are thought to be transported through bulk flow [13,19]. Other secreted proteins have been shown to require cargo receptors for efficient ER-to-Golgi transport [14,15].

While certain ER chaperones, such as GRP78 and PDI, have been observed to leave the ER and localize to the cell surface, especially during ER stress [20], their primary roles lie in assisting protein folding and ensuring quality control within the ER. Following their escape from the ER, the majority of ER resident proteins containing the KDEL retention signal are returned to the ER via COPI vesicles, facilitated by the KDEL receptor, which acts as a retrograde transport receptor [21]. Additionally, specific ER membrane proteins, such as TANGO1 and cTAGE5 from the MIA-family proteins, play a crucial role in the sorting and packaging of bulky cargo at the ER exit sites (ERES) by bridging the cargo and the COPII coat through interactions with the HSP47 chaperone in the ER lumen and SEC23/SEC24 in the cytosol [22,23]. Although TANGO1 was initially identified as a cargo receptor for collagens, recent studies indicate that its primary function lies in organizing the ERES [24,25]. Recent developments in understanding receptor-mediated ER-to-Golgi transport have focused on two prototypical cargo receptors, the LMAN1–MCFD2 complex and SURF4, which will be discussed in the following sections.

## Structure and function of the ER-Golgi intermediate compartment

**Figure 1.**
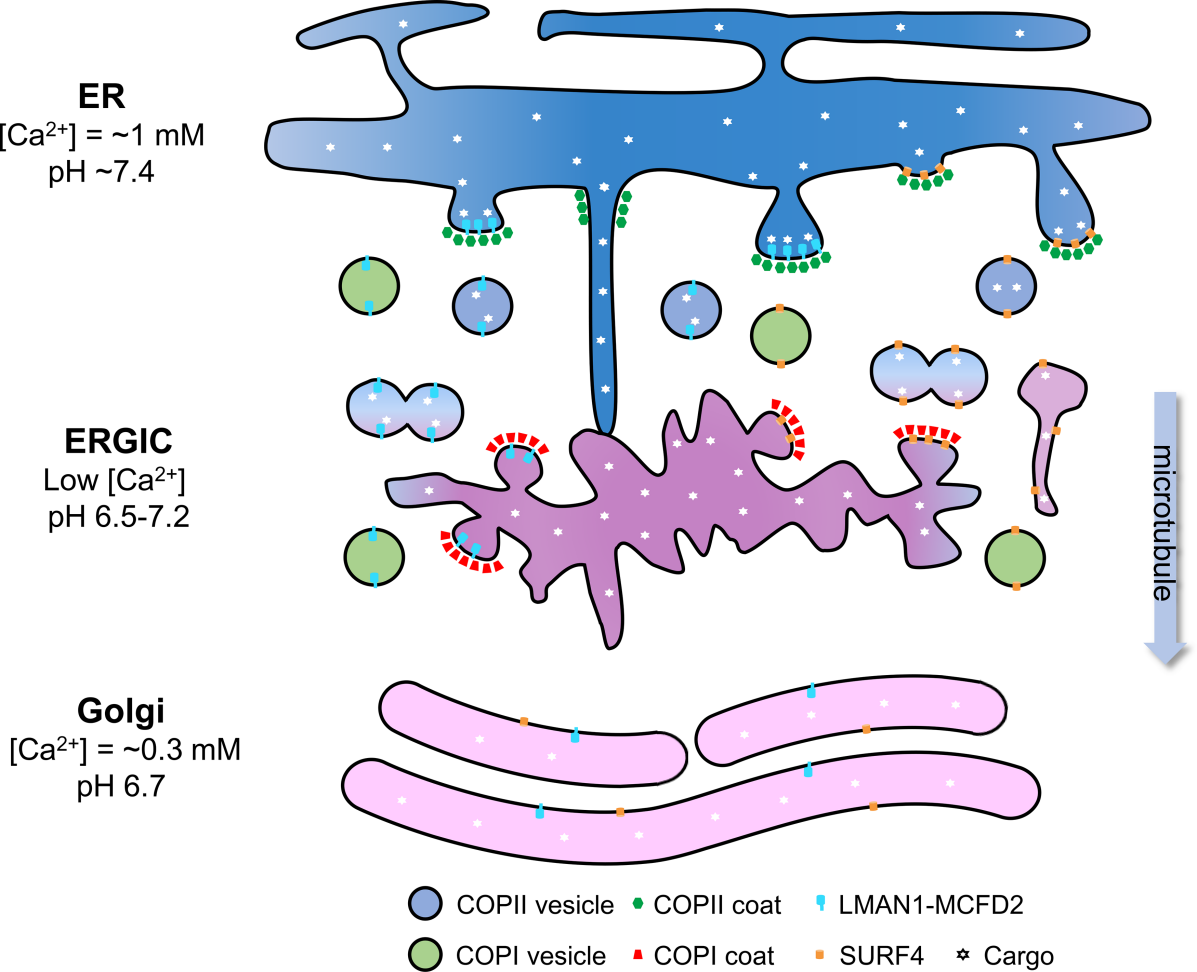
The LMAN1–MCFD2 complex and SURF4 as cargo receptors that facilitate the ER exit of secretory proteins. The vast majority of proteins leave the ER in COPII-coated vesicles at ER exit sites. Soluble cargo proteins are loaded into these vesicles either by binding to transmembrane transport receptors or through bulk flow. Additionally, cargo receptors can impact the morphology of ER exit sites. Some cargo proteins may bypass spherical COPII vesicles and instead utilize COPII-mediated tubules to travel directly to the ERGIC and *cis*-Golgi. The ERGIC is a dynamic organelle created by homotypic fusions of COPII vesicles, which can feature sub-regions with unique structures specialized for specific cargo receptors, such as SURF4. After arriving at the ERGIC or Golgi along microtubules, cargoes are unloaded, while cargo receptors return to the ER in COPI vesicles. The ER lumen has a neutral pH and higher Ca^2+^ concentration, in contrast with the acidic environment and lower Ca^2+^ concentrations found in the ERGIC and Golgi. Cargo loading and unloading can be impacted by changes in luminal pH between ER and Golgi and may also be influenced by Ca^2+^ and other factors. LMAN1 can sometimes escape the ER retention mechanism and reach the cell surface. This trafficking process is important in transporting several exogenous proteins and viruses.

In mammalian cells, a homotypic fusion of COPII vesicles creates the ER-Golgi intermediate compartment (ERGIC), a distinct tubulovesicular structure (Figure 1). This organelle is defined by the marker protein LMAN1, also known as ERGIC-53, and remains unabsorbed into the ER upon brefeldin A treatment. Often observed near the ERES and in the vicinity of the Golgi ribbon, ERGIC is the major sorting station in the early secretory pathway, where ER resident proteins are constantly recycled back to the ER in COPI vesicles [26,27]. Cargo proteins are also thought to dissociate in the ERGIC, where they continue to the *cis*-Golgi network via microtubules while the receptors are returned to the ER for further rounds of cargo transport. TANGO1 is important in maintaining structures of the early secretory machineries. TANGO1 knockout disrupts the ERGIC, leads to mislocalization of LMAN1 and SURF4, and impairs the secretion of both large and small soluble proteins [24]. The structure of ERGIC is dynamic and may be influenced by the inclusion of specific cargo receptors [28]. Loss of SURF4 leads to a reduction in the number of COPII-positive ERES, indicating that SURF4 is involved in organizing the ERES [29]. It has been observed that ERES structures are highly dynamic, able to form tubular extensions connecting the ER with the ERGIC/Golgi [30]. Highly elongated tubular ERGIC structures are involved in the selective and efficient transport of soluble cargoes of SURF4 [31]. These structures are devoid of LMAN1 yet positive for Rab1A/B, indicating that ERGIC morphology and function can be shaped by transporting cargo (Figure 1). Apart from its central role in ER-Golgi trafficking, ERGIC also serves as a significant source of membranes for packaging bulky cargo at ERES [32] and for the formation of autophagosomes [33]. Coronaviruses, including SARS-CoV-2, typically hijack and modify host cell secretory membranes. During viral assembly, the genomic RNA-nucleocapsid protein complex of coronaviruses is enveloped by the membrane of the ERGIC, where viral membrane proteins are embedded [34]. This process shares similarities with the formation of intraluminal vesicles in endosomes, but its mechanism remains unknown despite the potential for therapeutic intervention.

## The LMAN1–MCFD2 complex

**Figure 2.**
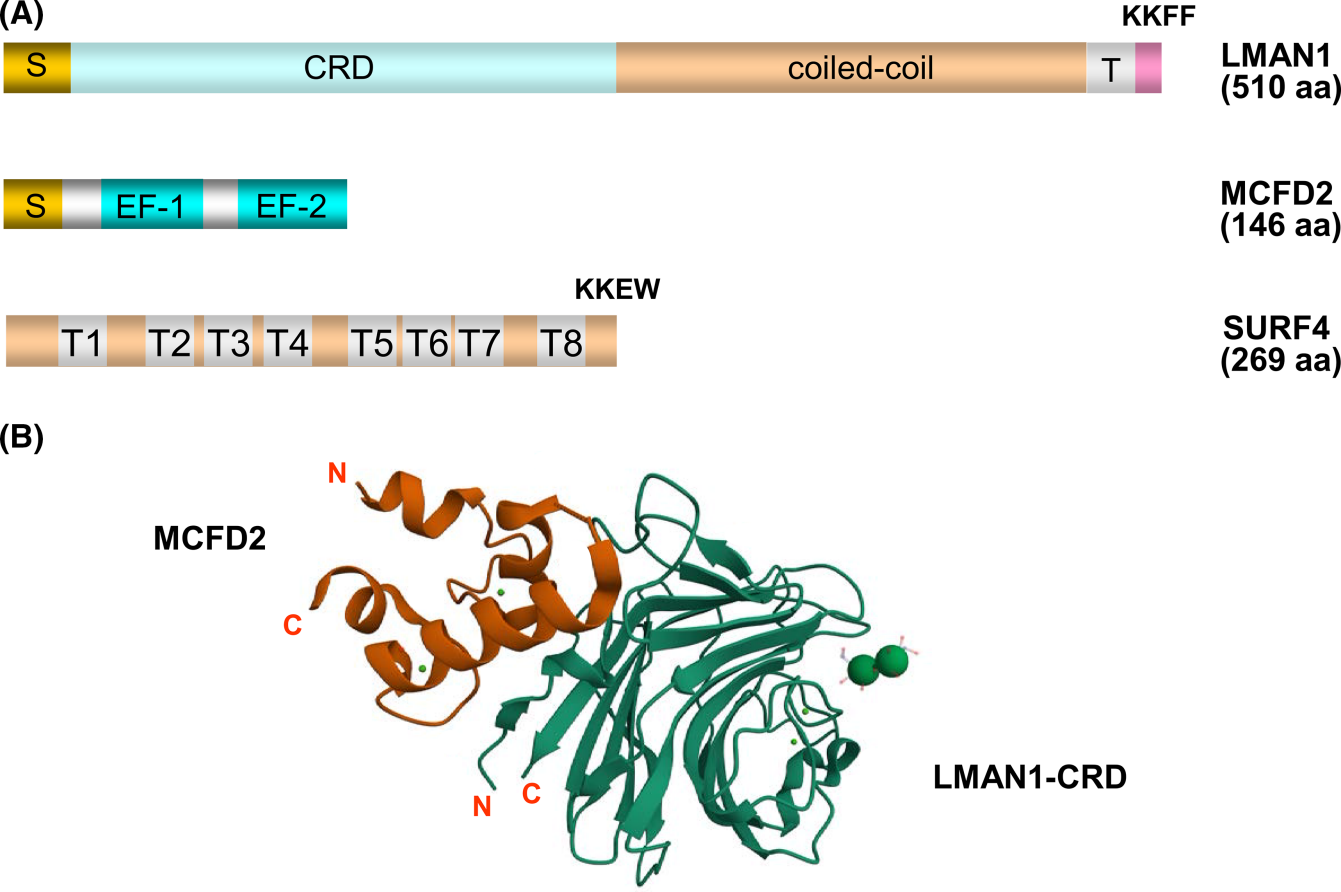
Functional domains and structures of LMAN1/MCFD2 and SURF4. (**A**) Functional domains of LMAN1, MCFD2, and SURF4. S, signal sequence; T, transmembrane domain. The C-terminal sequences of LMAN1 and SURF4 are shown to indicate ER retrieval (KK) and exit (FF) signals. (**B**) Crystal structure the CRD of LMAN1 bound to mannobiose (Man-α1,2-Man) in complex with MCFD2 in the presence Ca^2+^ (PDB ID: 3WHU) [51]. The transmembrane topology of SURF4 was predicted using the DeepTMHMM program. Large green spheres represent mannoses and small green spheres represent Ca^2+^. An AlphaFold structural model of SURF4 can be found in ref. [80].

LMAN1 is a 53 kDa type I transmembrane protein that forms a homo-hexameric complex and belongs to the family of L-type animal lectins (Figure 2). It is highly conserved among vertebrates, with 87.5% sequence identity between *Homo sapiens* and *Mus musculus*. Clear authologs in lower eukaryotes such as *C. elegans* and *Drosophila*. The luminal segment of LMAN1 contains a carbohydrate recognition domain (CRD) and a putative coiled-coil domain [35]. The sugar-binding pocket of the CRD also exist is held by two Ca^2+^ and specifically binds high-mannose oligosaccharides [36,37]. The coiled-coil domain is required for protein oligomerization [38]. The short C-terminal cytosolic tail contains a dilysine (KK) motif, which is necessary for retrograde transport in COPI vesicles, and a diphenylalanine (FF) motif required for ER export in combination with the coiled-coil domain and the transmembrane domain [39]. The KKFF motif results in the constitutive recycling of LMAN1 between the ER and Golgi (Figure 1). LMAN1 undergoes a process of concentrative sorting by the COPII coat, and a dimeric FF–FF motif generated by the oligomerization of LMAN1 is required for its interaction with COPII [40]. Therefore, a LMAN1 dimer represents the minimal unit for ER exit. LMAN1 was speculated to be a cargo receptor since its discovery and it was later found to be required for the secretion of lysosomal proteins cathepsin C [41] and cathepsin Z [42]. Genetic studies identified mutations in *LMAN1* as the cause for the combined deficiency of coagulation factor V (FV) and factor VIII (FVIII) (F5F8D) [43], which is an autosomal recessive bleeding disorder with both FV and FVIII in 5–30% of normal [44].

Although LMAN1 exhibits characteristics of a cargo receptor, it is insufficient for the transport of FV and FVIII cargo. Approximately 30% of individuals with F5F8D carry mutations in a second gene called *MCFD2* [45]. MCFD2 is also highly conserved among vertebrates, with 82.2% sequence identity between *Homo sapiens* and *Mus musculus*. However, no clear authologs can be identified in lower eukaryotes. LMAN1 and MCFD2 form a Ca^2+^-dependent complex with 1 : 1 stoichiometry [46] and MCFD2 deficiency does not affect the level and localization of LMAN1. MCFD2 is a 16 kDa soluble monomeric protein (Figure 2). Except for a disordered N-terminal sequence, the majority of the protein consists of two calmodulin-like Ca^2+^-binding motifs known as EF-hand domains [45,47]. MCFD2 lacks an ER retrieval signal and is localized to the ERGIC by binding to LMAN1 [45,46]. In the absence of LMAN1, MCFD2 is secreted out of cells. Ca^2+^ binding is critical for overall MCFD2 tertiary structure formation. Without Ca^2+^, MCFD2 assumes a disordered structure [47]. The crystal structure analysis of LMAN1–CRD in complex with MCFD2 revealed that the EF-hand domains of MCFD2 interact with the N-terminal region of LMAN1 (Figure 2B) [48–51]. Mutations at the N-terminus of LMAN1 disrupt MCFD2 binding without affecting mannose-binding [52]. Conversely, mutations abolishing the mannose-binding of LMAN1 do not affect MCFD2 binding, suggesting that LMAN1 has distinct and separable binding sites for both MCFD2 and high-mannose glycans present in its cargo [52]. Even though a complex between monomeric LMAN1–CRD and MCFD2 can be readily formed *in vitro*, monomeric LMAN1 is not capable of interacting with MCFD2 and is defective in exiting the ER *in vivo*. [52].

## Cargoes that are transported by the LMAN1–MCFD2 complex

FV and FVIII are the most relevant physiological cargoes in mammals, as evidenced by decreased plasma levels of these proteins in patients with F5F8D [44] and mice with LMAN1 or MCFD2 deficiency [53,54]. In addition, male LMAN1 or MCFD2 knockout (KO) mice show a mild decrease in plasma α1-antitrypsin (AAT) levels [54], consistent with the identification of AAT as a LMAN1 cargo [55] and confirming the requirement for MCFD2. FV and AAT accumulate in the ER of hepatocytes in LMAN1 KO, MCFD2 KO, and double KO mice [53,54], suggesting an ER exit defect for these proteins. Further studies showed that endogenous AAT secretion decreased and intracellular AAT levels elevated in LMAN1 and MCFD2 KO HepG2 cells due to a delayed ER-to-Golgi transport of AAT [56]. Surprisingly, secretion of the highly polymerogenic AAT Z (E342K) variant is also LMAN1-dependent, similar to the wild-type AAT [56]. An independent study identified *LMAN1* as a gene affecting the trafficking of another polymerogenic AAT King's variant (H334D) using a genome-wide CRISPR screening [57].

Both LMAN1 and MCFD2 are widely expressed in various tissues, indicating that they are involved in the transport of diverse cargo in addition to FV, FVIII, and AAT. Previous studies have identified cathepsin C and cathepsin Z as LMAN1 cargo in cell-based experiments [41,42], but there was no significant reduction in the levels of these proteins observed in liver extracts of LMAN1 knockout mice [53]. Therefore, the physiological relevance of these cargoes remains unclear. LMAN1 has been shown to transport other cargo proteins, such as Mac-2 binding protein (Mac-2BP) [58], γ-aminobutyric acid type A receptors (GABAARs) [59], and matrix metalloproteinase-9 (MMP-9) [60]. In the case of GABAARs, LMAN1 KO led to decreased protein levels in the mouse brain [59]. LMAN1 plays a role in facilitating the transport of ERp44, a protein involved in post-ER quality control, to the ERGIC and Golgi, where ERp44 binds its client proteins for retrieval back to the ER [61,62]. LMAN1 also assists in the assembly and transport of IgM polymers by interacting with both ERp44 and polymerized IgM [63,64]. In addition, LMAN1 is involved in photoreceptor transport and homeostasis, as evidenced by abnormal levels and locations of Rhodopsin in LMAN1 deficient mice [65,66]. Both LMAN1 and MCFD2 play a role in transporting FV, FVIII, AAT, ERp44/IgM, and Mac-2BP, and it is likely that the majority of cargoes require the synergistic actions of both proteins. However, it is uncertain whether this applies to all LMAN1-dependent cargoes. Notably, LMAN1 KO mice exhibit a strain-specific partial lethal phenotype [53], which is absent from MCFD2 KO mice [54], suggesting that there are unidentified LMAN1-specific cargoes or other LMAN1-specific functions distinct from those of MCFD2.

## Mechanism of cargo transport by the LMAN1–MCFD2 complex

Since the majority of cargoes for the LMAN1–MCFD2 complex identified to date are glycoproteins, LMAN1 is thought to interact with the N-glycans on the cargo. FV and FVIII share a similar domain structure (A1–A2–B–A3–C1–C2), with the A and C domains displaying strong sequence homology. Although the B domains are highly divergent between the two proteins, they are both heavily glycosylated. The binding of B-domain deleted FVIII to the LMAN1–MCFD2 complex is markedly reduced, suggesting that the interaction of the CRD with N-glycans in the B domain plays an important role in cargo binding [46,67]. This is further supported by the observation that the interaction between FVIII and LMAN1 mutants with carbohydrate-binding defects was decreased [68]. However, these mutant LMAN1 can still partially rescue FVIII secretion [69], and cross-linking of FVIII with LMAN1 can be observed in cells treated with the N-glycosylation inhibitor tunicamycin [46]. Although N-glycan binding of LMAN1 is essential for the secretion of Mac-2BP, the interaction of LMAN1 with unglycosylated Mac-2BP was still observed [58]. Similarly, both GABAARs and MMP-9 interact with LMAN1 in a glycan-independent manner [59,60]. In addition, as a LMAN1 cargo, ERp44 is not a glycoprotein. These observations suggest that LMAN1 recognizes cargo proteins not only through N-glycan binding but also through direct protein–protein interaction. This result may also explain why no missense mutations have been identified in the carbohydrate-binding pocket of LMAN1 [44]. Overexpression of both LMAN1 and MCFD2 did not enhance FVIII secretion, indicating that the receptor complex expression is not a limiting factor in cargo transport [69]. Mice carrying a hypomorphic *Lman1* allele that expresses 6% to 8% of WT *Lman1* mRNA levels exhibit ∼70% of WT levels for both FV and FVIII [70], indicating that even low levels of LMAN1 may be adequate for FV/FVIII secretion.

All MCFD2 missense mutations in F5F8D patients are located in the EF-hand domains and abolish LMAN1 binding [52], indicating that the LMAN1–MCFD2 complex formation is required for FV and FVIII transport. The requirement of a transmembrane component (LMAN1) and a soluble cofactor (MCFD2) is a unique characteristic of this cargo receptor, suggesting a more sophisticated mechanism for cargo trafficking in higher eukaryotes. In both humans and mice, MCFD2 deficiency is associated with slightly lower plasma levels of FV and FVIII than LMAN1 deficiency [44,54]. Interestingly, FV and FVIII levels in double KO mice match the higher levels found in LMAN1 KO mice [54]. Overexpression of MCFD2 can rescue FV/FVIII secretion of LMAN1 KO cells, whereas overexpression of LMAN1 has no effect on FV/FVIII secretion in MCFD2 KO cells [69]. The interaction between MCFD2 and FV/FVIII does not depend on LMAN1, but both interactions involve the EF-hand domains [71], suggesting that MCFD2 likely functions as a primary interacting partner of FV/FVIII cargo. However, under physiological conditions, the LMAN1-cargo interaction is likely necessary for efficient cargo transport out of the ER. Evidence supporting this includes the detection of AAT-LMAN1 interaction in MCFD2 KO cells [56] and unchanged interactions of cathepsins with LMAN1 in MCFD2 knockdown cells [72]. The interaction of LMAN1 with MCFD2 not only enhances the sugar-binding ability of LMAN1 [37], but also induces significant conformational changes in MCFD2 [48–51]. The binding of LMAN1 may allosterically activate MCFD2 to bind polypeptide segments of cargo proteins [50]. Therefore, it is likely that AAT and other cargoes contain dual sorting signals that are recognized sequentially by MCFD2 and LMAN1 for efficient cargo transport, potentially through conformation-based signals [73]. Among potential cargoes of the LMAN1–MCFD2 complex, no common sequence motifs are obvious, suggesting that MCFD2 may recognize diverse sorting signals. MCFD2 is thought to exhibit significant conformational flexibility to accommodate various polypeptide ligands [50]. A recent study proposed a putative common MCFD2-binding motif (SDLLMLLRQS) located in the B domains of FV and FVIII based on the homology between these proteins [74], but a subsequent study found that deletion of this motif does not affect FV/FVIII secretion [69].

## Other potential functions of LMAN1 and MCFD2

LMAN1 is a component of the early secretory pathway that can sometimes escape the ER retention mechanism and reach the cell surface (Figure 1). Recent research has shown that LMAN1 on the cell surface of dendritic and airway epithelial cells serves as a receptor for house dust mite (HDM) allergens [75]. LMAN1 binds mannosylated HDM allergens via the CRD. After internalization, LMAN1 down-regulates the activation of the NF-kB pathway in response to HDM or other inflammatory stimuli. In fact, a decrease in LMAN1 expression is associated with the asthmatic disease state [75]. Cell-surface trafficking of LMAN1 is also involved in transporting pathogen proteins. Cholix, which is secreted by the intestinal pathogen Vibro cholera, undergoes transcytosis from apical vesicles to the basolateral region of cells by interacting with LMAN1 [76,77]. LMAN1 plays a crucial role in the propagation of several highly pathogenic RNA and DNA viruses. LMAN1 binds to virus glycoproteins, traffics to virus budding sites of the plasma membrane, and is incorporated into virions, essential for the formation of infectious virus particles [78]. Depletion of LMAN1 results in the production of noninfectious virus particles for several RNA viruses such as arenavirus, coronavirus, and filovirus [78]. LMAN1 up-regulation was observed after a murine gammaherpesvirus infection, and inhibition of LMAN1 resulted in a significant reduction in virus production [79], suggesting that LMAN1-dependent secretory pathway is rate-limiting for virus production. Moreover, LMAN1 interacts with the N146-glycan of the hepatitis B virus (HBV) envelope and is crucial for HBV viral particle release [80]. Silencing of LMAN1 inhibited HBV virion egress without affecting HBV noninfectious spherical subviral particle secretion. The involvement of LMAN1 in these non-canonical trafficking events highlights its versatility and importance in various cellular processes and could present potential targets for pharmacological interventions beyond traditional cargo transport mechanisms. Whether MCFD2 plays a role in these processes remains to be determined.

## SURF4

SURF4 is the metazoan ortholog of the yeast Erv29p, with ∼30% sequence homology between human and yeast proteins. Among mammalian species, SURF4 is extremely well conserved, with 99.3% sequence identity between *Homo sapiens* and *Mus musculus* [81]. SURF4 is a protein with eight predicted transmembrane domains and a C-terminal dilysine ER retrieval motif that interacts with the COPI coat (Figure 2A). It serves as a cargo receptor for several yeast proteins by interacting with them through the I–L–V motif [82]. Although SURF4 has been speculated to be a mammalian cargo receptor for a long time, its client cargoes have only recently been identified. These include apolipoprotein B (ApoB) [29] and a series of proteins with the so-called ER-Exit by Soluble Cargo using Amino-terminal Peptide-Encoding (ER-ESCAPE) motif immediately after signal sequences [83]. The ER-ESCAPE motif consists of three amino acid residues (hydrophobic-Proline-hydrophobic) that interact with SURF4. A genome-wide CRISPR–Cas9 screening identified PCSK9 as a SURF4 cargo [84]. This finding explains why the secretion of PCSK9 is defective in SEC24A-deficient mice [6]. SURF4 interacts with the B-site of SEC24A and SEC24B, bridging soluble cargoes in the ER lumen with the COPII coat on the cytosolic side [85]. In contrast, LMAN1 interacts with all four paralogs of SEC24 [86]. Other SURF4 cargoes include erythropoietin [87], polymeric AAT [57], sonic hedgehog (Shh) [88,89], proinsulin [90], and prosaposin [91]. Although PCSK9 does not have the ER-ESCAPE motif immediately after signal peptide cleavage, self-cleavage of the pro-domain in the ER exposes an IPW tripeptide as a signal [85]. SURF4 also recognizes additional signals in cargo that do not contain the ER-ESCAPE motif. One such signal is the polybasic motif (BBBXXBB, where B represents a basic amino acid) in Shh and other secretory proteins [88,89]. Yet some SURF4 cargo proteins, such as ApoB, erythropoietin, and proinsulin, contain no ER-ESCAPE or polybasic motifs. In addition, both LMAN1 and SURF4 can transport polymerogenic King's variant of AAT [57], suggesting AAT polymers display sorting signals for two different cargo receptors. The nature of additional sorting signals recognized by SURF4 is unclear. SURF4 likely transports a cargo that's required for early embryonic development, as SURF4-deficient mice die between embryonic days 3.5 and 9.5 [92]. Hepatocyte-specific KO of *Surf4* resulted in decreased plasma PCSK9 and ApoB levels, and increased low-density lipoprotein receptor abundance [93,94]. The combined effects of decreased plasma PCSK9 and ApoB led to a marked reduction in blood cholesterol and triglyceride levels. Intestinal-specific *Surf4* KO mice exhibited lipid accumulation in enterocytes and impaired fat absorption and secretion [95].

## The release of cargo in the ERGIC and Golgi

Cargo release in the ERGIC and Golgi was thought to be triggered by a decrease in luminal pH (Figure 1), analogous to the release of endocytic cargo in endosomes [96]. A histidine residue in LMAN1 was identified as a potential proton sensor [96]. Subsequent research suggests that Ca^2+^ may also play an important role in the release of cargo [68]. Evidence showed that the luminal Ca^2+^ concentration in the ERGIC is much lower than in the ER [97,98]. The stable, Ca^2+^-dependent LMAN1–MCFD2 complex remains intact through multiple rounds of cargo capture and release [45,46], suggesting that this protein complex is insensitive to changes in Ca^2+^ concentrations from the ER to the ERGIC. Indeed, low Ca^2+^ concentrations disrupted the interaction of the LMAN1-CRD with 2,3-mannobiose without dissociating the LMAN1–MCFD2 complex [68]. Structural studies suggest that LMAN1 is apt to lose Ca^2+^ ions in comparison with MCFD2 [51]. Therefore, in the low Ca^2+^ concentration environment of the ERGIC, the cargo proteins could be released from the LMAN1–MCFD2 complex due to disruption in N-glycan–LMAN1 interaction, allowing the intact LMAN1–MCFD2 receptor complex to recycle back to the ER via COPI vesicles to undergo additional rounds of cargo transport [99]. Other mechanisms may also regulate the release of cargo. SURF4 was shown to bind and carry Shh to the *trans*-Golgi, where proteoglycans (PG) compete with SURF4 to interact with the polybasic motif in Shh, providing a SURF4-to-PG relay mechanism for dissociating cargo proteins from the SURF4 cargo receptor [88]. As a zinc-binding protein, ERp44 dissociation from LMAN1 is triggered by the higher Zn^2+^ and lower pH environment in the Golgi [62].

## Conclusions

The receptor-mediated ER-Golgi transport model stipulates that specific signals are embedded in cargo proteins that are recognized by their corresponding receptors. Therefore, the identification of such signals is crucial for confirming the pairing of cargo and receptor. Whereas some sorting signals in SURF4 have been recently identified, the specific cargo signals recognized by the LMAN1–MCFD2 complex prove to be elusive. Additionally, SURF4 is capable of transporting various cargoes with unidentified binding motifs. Elucidating how cargo receptors recognize such a diverse set of cargo proteins is critical for understanding the general mechanism of receptor-mediated cargo transport. Although recent studies have provided new insights into the actions of cargo receptors and the structure and function of the ERGIC, many questions remain unanswered. With thousands of soluble proteins traversing the early secretory pathway, it is likely that additional cargo receptors exist but have yet to be discovered and characterized. In cases where deficiency in a cargo receptor results in only mild to moderate decreases in the secretion of a specific cargo, it is uncertain whether the balance of secretion is due to bulk flow or complementation by other cargo receptors with overlapping functions. Exploring the mechanism through which cargoes locate cargo receptors and become detached from the congested ER milieu, as well as how cargoes are discharged in the ERGIC/*cis*-Golgi, is a crucial area for future research. Additionally, it's worth mentioning that the transportation of medically significant proteins by cargo receptors and the unconventional trafficking of pathogen proteins and viruses may present promising therapeutic targets.

## Perspectives

- How proteins are transported from the ER to the Golgi is not fully understood. At least a portion of soluble secretory proteins is thought to display sorting signals that are recognized by transmembrane cargo receptors. Some soluble secretory proteins are believed to exhibit sorting signals that are identified by transmembrane cargo receptors, although only a limited number of cargo receptors have been identified.
- Recent investigations into two mammalian cargo receptors, the LMAN1–MCFD2 complex and SURF4, have identified several cargo proteins transported by these receptors, along with two cargo sorting signals for SURF4 and mechanisms of action (in terms of the LMAN1–MCFD2 complex, the separate roles of the two subunits).
- A better understanding of the mechanisms underlying receptor-mediated cargo transport could potentially result in new treatments for diseases caused by early secretory pathway defects.

## Open Access

Open access for this article was enabled by the participation of Cleveland Clinic in an all-inclusive *Read & Publish* agreement with Portland Press and the Biochemical Society under a transformative agreement with Individual.

## Abbreviations

AAT: α1-antitrypsin
AMELX: amelogenin X-linked
COPI: coat protein complex I
COPII: coat protein complex II
CRD: carbohydrate recognition domain
DSPP: dentin sialophosphoprotein
ER: endoplasmic reticulum
ERES: ER exit sites
ER-ESCAPE: ER-Exit by Soluble Cargo using Amino-terminal Peptide-Encoding
ERGIC: ER-Golgi intermediate compartment
F5F8D: combined deficiency of FV and FVIII
FV: coagulation factor V; coagulation FVIII factor VIII
GABAARs: γ-aminobutyric acid type A receptors
HBV: hepatitis B virus
HDM: house dust mite
KO: knockout
Mac-2BP: Mac-2 binding protein
MMP-9: matrix metalloproteinase-9
PG: proteoglycans
WT: wild-type
